# Assessment of Trace Element Levels in Muscle Tissues of Fish Species Collected from a River, Stream, Lake, and Sea in Sakarya, Turkey

**DOI:** 10.1155/2014/496107

**Published:** 2014-03-24

**Authors:** Tülay Küpeli, Hüseyin Altundağ, Mustafa İmamoğlu

**Affiliations:** Chemistry Department, Faculty of Arts and Science, Sakarya University, 54187 Sakarya, Turkey

## Abstract

Levels of some trace and essential elements, including Al, B, Ba, Cr, Cu, Fe, Mn, Ni, Sr, and Zn, were determined in 17 different fish species from Sakarya River, Çark Stream, Sapanca Lake, and Western Black Sea using ICP-OES after microwave (MW) digestion procedure. During preparation of samples for analysis, wet and MW digestion methods were also compared. Accuracy of the digestion methods was checked by the analysis of DORM-3 reference material (Fish Protein Certified Reference Material for Trace Metals). Concentrations of trace elements were found as Al: 6.5–48.5, B: 0.06–3.30, Ba: 0.09–2.92, Cr: 0.02–1.64, Cu: 0.13–2.28, Fe: 7.28–39.9, Mn: 0.08–11.4, Ni: 0.01–26.1, Sr: 0.17–13.5, and Zn: 11.5–52.9 *µ*g g^−1^. The obtained results were compared with other studies published in the literature. Trace element levels in various fish species collected from waters in Sakarya region were found to be below limit values provided by Turkish Food Codex (TFC), Food and Agriculture Organization (FAO), and World Health Organization (WHO).

## 1. Introduction

It is significantly important to determine and monitor heavy metal levels in foodstuffs, particularly seafood, because heavy metal ions can easily accumulate in such food compared to other foodstuffs and cause harmful effects on human health [1, 2]. Discharge of industrial waste water without a pretreatment into lakes, rivers, stream, and sea primarily causes an increase in heavy metal ion concentration in such water environments. Moreover, such waters are speedily polluted by chemical substances, paints, petroleum products, and industrial, domestic, and modern agriculture wastes [3, 4]. Metal pollutants in the form of particles, metal ions, and organic and inorganic compounds in water also toxify the associated ecosystem [5, 6]. Potential of heavy metal ions to accumulate in seafood, including fish living in waters polluted with heavy metals, is rather high. Heavy metals, which especially accumulate in organs of fish, such as internal organs, kidneys, and spleen, can be transmitted to and accumulated in various organs of human body by their consumption [5, 7]. Therefore, introduction of heavy metals into food chain threatens human health. On the other hand, fish is one of the most important foods to be eaten for a healthy life because it has a high protein quality and nutritional value [8]. However, it is suggested worldwide that fish and seafood are consumed more in order to prevent cardiovascular diseases and some other diseases. Fish is especially recommended to infants, elderly, cardiac patients, those who had a brain hemorrhage, and those experiencing digestion problems because it has high mineral content and low energy level [9]. Although consumption of fish and seafood is useful for human health, it may cause toxic effects by transmission into human body by food of heavy metal ions as a result of accumulation in fish's body of pollutants that may be present in water and cause a risk for human health [10, 11].

Although Turkey is surrounded on three sides by the sea, the amount of seafood consumed per capita is low and mainly concentrates in coastal regions. However, despite this, fish consumption has increased in recent years [12]. Annual average amount of seafood consumed per capita was 5 kg until 2004 and increased in 2012 by 80% to 9 kg [13], which is still below 12 kg per capita recommended by WHO [14].

In this study, levels of some trace elements, including Al, B, Ba, Cr, Cu, Fe, Mn, Ni, Sr, and Zn, were measured by ICP-OES in 17 different fish species caught in the waters of Sakarya River, Çark Stream, Sapanca Lake, and Western Black Sea during September and October, 2012. Wet and MW digestion techniques were compared for preparation of fish samples to measurement step by ICP-OES. Accuracy of these methods was checked by the analysis of DORM-3, a certified reference material (Fish Protein Certified Reference Material for Trace Metals). The results were discussed by comparing with previous studies reported in the literature as well as standard values in Turkish Food Codex (TFC), Food and Agriculture Organization (FAO), and World Health Organization (WHO).

## 2. Material and Method 

### 2.1. Preparation of Samples for Analysis

**Figure 1:**
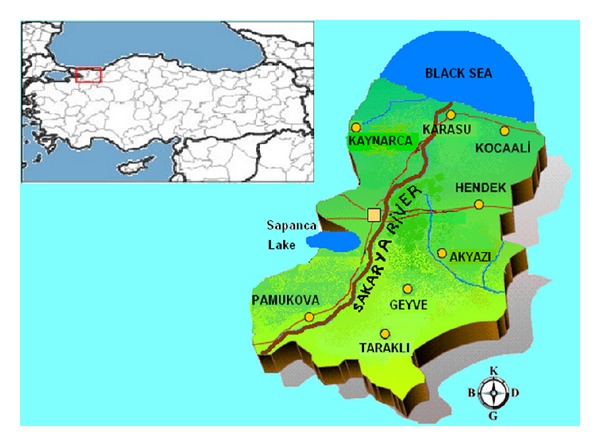
The map showing the sampling area.

24 samples were collected from 17 different fish species caught in the waters of Sakarya River, Çark Stream, Sapanca Lake, and Western Black Sea within the borders of the province of Sakarya during September and October, 2012. Fish species were* Silurus glanis*,* Blicca bjoerkna*,* Capoeta pestai*,* Cyprinus carpio*,* Scardinius erythrophthalmus*,* Mugil cephalus*,* Barbus capito*,* Esox lucius*,* Tinca tinca*,* Trachurus mediterraneus*,* Sarda sarda*,* Mullus barbatus*,* Engraulis encrasicolus*,* Gobius niger*,* Merlangius merlangus*,* Belone belone*, and* Pomatomus *saltatrix. Internal organs, head, tail, and scales of fish samples were cleaned using a stainless-steel knife; muscle tissues obtained were washed with ultradistilled water and dried using filter paper. The samples were dried in drying oven at 110°C for approximately 4 hours, homogenized in a porcelain mortar, and kept in polyethylene bags at −20°C until the day of analysis. The studied area are shown in Figure 1.

### 2.2. Reagents

Glass and plastic containers used in the study were allowed to stand in a 10% HNO_3_ solution overnight and rinsed using ultradistilled water (Milli-Q Millipore 18.2 MΩ·cm). All reagents used in the analysis are analytical or of higher grade. Suprapur nitric acid (65%, w/w), suprapur hydrochloric acid (37%, w/w) and analytical grade hydrogen peroxide (30%, w/w), were purchased from Merck (Germany). All solutions used in the study were prepared using ultradistilled water. ICP multielement standard solution of 1000 mg L^−1^ supplied by Merck was used after step-by-step dilution. Accuracy and precision of the results of the samples were checked using standard reference material (CRM, DORM-3) supplied from the National Research Council of Canada (CNRC).

### 2.3. Instruments

ICP-OES (inductively coupled plasma-optical emission spectrometer, Spectro Analytical Instruments, Kleve, Germany) was used for the determination of concentration of elements. The experimental conditions used on ICP-OES are given in Table 1. Milestone Ethos D MW closed system (maximum pressure 1450 psi, maximum temperature 300°C) was used to digest fish muscle samples, whereas IKA RCT classic model heater with magnetic stirrer was used to digest samples by wet decomposition procedure.

### 2.4. Sample Digestion Procedure

In this study, wet and MW digestion techniques for fish samples were compared. In both methods, 0.25 g of reference material and 1 g of fish sample were employed in duplicate.

In MW digestion procedure, 6 mL of 65% HNO_3_ solution and 2 mL of 30% H_2_O_2_ solution were added to the samples and the samples were dissolved in MW system. A program of 32 minutes was used during digestion procedure (2 min, 250 W, 100°C; 2 min, 250 W, 100°C; 5 min, 300 W, 120°C; 5 min, 550 W, 170°C; 6 min, 700 W 200°C; waiting period: 12 min). At the end of the procedure, the solutions were completed to a volume of 10 mL using ultradistilled water.

In wet digestion procedure, 6 mL of 65% HNO_3_ solution and 2 mL of 30% H_2_O_2_ solution were added to a sample of 1 g and the samples were dissolved at 130°C for 4 hours by heating [8]. After the samples were evaporated until they come to an appropriate dryness, they were filtered using filter paper and completed to a volume of 10 mL using ultradistilled water.

### 2.5. Validation of the Method

Performance of the method was evaluated in terms of linearity, recovery, and precision. The results obtained for concentrations of Al, B, Cu, Cr, Co, Fe, Mn, Ni, Zn, Cu, Cr, Fe, Ni, and Pb in Fish Protein Certified Reference Material for Trace Metals (DORM-3) were used to validate the method.

## 3. Results

Levels of the studied elements were given in dry basis after determination of their concentration by ICP-OES. In order to determinate and compare the digestion procedures including MW and wet, DORM 3 reference material was used. The recoveries of both methods were calculated. The obtained and certified results are presented in Table 2. Recovery values by MW technique are in the range from 95 to 98%, whereas recovery values by wet digestions are in the range from 92 to 95%. MW technique provided higher and more accurate recovery values for DORM 3 reference material. In order to see the performance of both techniques in real fish samples, muscle tissues of* Silurus glanis *were digested by MW and wet method, and then trace element levels were determined and the obtained results are given in Table 3. It was found that MW digestion procedure also provides higher values than wet digestion method in real samples. Since MW technique provides an opportunity to work faster and allows lesser amount of analyte lost, it was chosen as the digestion method in this study.

Table 4 shows trace element concentrations in muscle tissues of 17 different fish species, namely,* Silurus glanis*,* Blicca bjoerkna*,* Capoeta pestai*,* Cyprinus carpio*,* Scardinius erythrophthalmus*,* Mugil cephalus*,* Barbus capito*,* Esox lucius*,* Tinca tinca*,* Trachurus mediterraneus*,* Sarda sarda*,* Mullus barbatus*,* Engraulis encrasicolus*,* Gobius niger*,* Merlangius merlangus*,* Belone belone* and* Pomatomus saltatrix.* Concentrations of trace elements were found as Al; 6.5–48.5, B; 0.06–3.30, Ba; 0.09–2.92, Cr; 0.02–1.64, Cu; 0.13–2.28, Fe; 7.28–39.9, Mn; 0.08–11.4, Ni; 0.01–26.1, Sr; 0.17–13.5, and Zn; 11.5–52.9* µ*g g^−1^.

### 3.1. Aluminium

Aluminium (Al) is found in large quantities in nature and introduced into living creatures through food chain, particularly through water [15]. Accumulation of Al in brain cells may cause Parkinson's disease and Alzheimer's disease [16]. The highest and lowest Al concentrations were determined in the fish species from Sapance Lake. The lowest Al concentration 6.50* µ*g g^−1^ was found in* Tinca tinca *species whereas the highest 48.5* µ*g g^−1^ in* Esox lucius *species. Al concentration in Turkey was reported as 0.02–5.41* µ*g g^−1^ in İskenderun Gulf and Northeastern Mediterranean [17]. An average Al concentration of 1.35* µ*g g^−1^ was found in a study on fish conducted in France [18]. Daily amount of Al entering a human body along with food in a person living in the city and weighing approximately 70 kg is 0.01–1.4 mg per kg of body weight [19].

### 3.2. Boron

Boron (B) is found in low concentrations in the form of compounds in soil, rocks, and water and introduced into human body by food since it cannot be synthesized in the body. It is released by disassociation of soil and sediments, arising from the environment and human beings. Relatively high concentrations of B are accepted as hazardous for human beings, water birds, and other wild living creatures [20]. The highest and the lowest B concentration in fish samples were 3.30* µ*g g^−1^ in* Blicca bjoerkna *species taken from Sakarya River and 0.06* µ*g g^−1^ in* Cyprinus carpio* species taken from Çark Stream, respectively. There was no information on B concentration in fish studies in the literature. Daily tolerable amount of boron in nutrition of human beings has been specified as 10–20 mg per 60 kg of body weight by FAO/WHO [21].

### 3.3. Barium

Barium (Ba) is found in the form of compounds, not in free form in nature. Ba ions are found in the waters of lakes, rivers, and stream because barium compounds easily dissolve in water. Thus, barium compounds can be absorbed by organisms living in water and accumulated in their bodies [22]. The lowest Ba concentration determined in fish species was found as 0.09* µ*g g^−1^ in* Cyprinus carpio *species taken from Sakarya River and the highest 2.92* µ*g g^−1^ in* Blicca bjoerkna *species taken from Çark Stream. Ba concentrations in fish species taken from Karasu were below detectable level. In a study by [23] performed in Turkey, Ba concentration was reported as 3.44–6.96* µ*g g^−1^. However, in a study by [18] performed in France and another one by [11], average Ba concentration was found as 0.065 mg kg^−1^and 0.003–2.8* µ*g g^−1^, respectively. Daily amount of barium recommended to be taken through food and drinks for an indiviual weighing 60 kg has been specified as 1.24 mg [24].

### 3.4. Chromium

Chromium (Cr) is considered as a basic trace element and found in human body, and it is known to control blood sugar and cholesterol level. Exposure of humans to Cr element is by inhalation, consumption of food containing Cr, and contact with Cr compounds [25]. According to our results, the lowest Cr concentration 0.02* µ*g g^−1^ was determined in* Barbus capito *species taken from Pamukova and the highest 1.64* µ*g g^−1^ in* Silurus glanis* species taken from Sakarya River. In general, it could be concluded that Cr level is low in muscle tissues of 17 fish species investigated in Sakarya. In a previous study, Cr concentration in fish was found in the range of 0.95–1.98* µ*g g^−1^ [26]. Cr concentration in canned fish was reported as 0.97–1.70* µ*g g^−1^ [27]. There is no information on the maximum Cr concentration in Turkish Food Codex [28]; however, the ratio of Cr recommended to be taken by a person of 60 kg was specified as 50–200* µ*g in the literature [29].

### 3.5. Copper

Copper (Cu) exists in the nervous system and has an important role during biological electron transfer. Furthermore, Cu is vital for synthesis of red blood cells. Cu deficiency may cause blood and nervous system diseases in adults [30]. The lowest Cu concentration 0.13* µ*g g^−1^ was found in* Cyprinus carpio *species taken from Pamukova and the highest in* Mullus barbatus *species taken from Karasu. Cu concentrations were determined as 0.51–7.05* µ*g g^−1^ in fish studies conducted in Aegean Sea and the Mediterranean [10]. Lower Cu concentrations (0.090–0.815* µ*g g^−1^) were found by [31]. Cu concentrations in fish species taken from Karasu compared with values of 1.09–2.75* µ*g g^−1^ were reported by [32]. Maximum Cu concentration allowed by Turkish Food Codex was specified as 20* µ*g g^−1^ [28]. Therefore, Cu concentration in muscle tissues in this study is below legal limits. Daily tolerable amount of Cu on the basis of body weight of 60 kg was determined as 3 mg by FAO/WHO [33].

### 3.6. Iron

Iron (Fe) deficiency is most commonly seen particularly in children and women in Turkey as worldwide and causes anemia. Fish is the most important source of Fe for adults and children [34]. At the end of the study, the lowest Fe concentration 7.28* µ*g g^−1^ was found in* Scardinius erythrophthalmus *species taken from Pamukova and the highest 39.9* µ*g g^−1^ in* Trachurus mediterraneus *species taken from Karasu. Higher values were previously found in fish in Turkey, 36.2–110* µ*g g^−1^ [35] and 5–70.1* µ*g g^−1^ [26]. However, lower values 0.78–4.21 and 1.55–6.71* µ*g g^−1^were found by [31, 36], respectively, in comparison to Fe concentrations in various fish species in Sakarya. According to Turkish standards, there is no information on maximum Fe concentration in fish samples [28]. Daily tolerable amount of Fe on the basis of body weight was determined as 48 mg by FAO/WHO [33].

### 3.7. Manganese

Manganese (Mn) is present in all tissues and a vital trace metal for functioning of many organic systems. Mn plays a major role in regulation of blood sugar and reproduction, digestion, and bone growth. Mn also acts as a cellular antioxidant [37]. The lowest Mn concentration of 0.08* µ*g g^−1^ in fish was found in* Barbus Carpito* taken from Pamukova,* Mullus barbatus* taken from Karasu and the highest Mn concentration of 11.4* µ*g g^−1^ in* Cyprinus carpio* species taken from Sakarya River. Reported Mn concentrations from fish studies in Turkey were 2.76–9.10* µ*g g^−1^ [35] and 1.00–9.40* µ*g g^−1^ [26]. Lower Mn concentrations were determined in studies carried out in Marmara, Aegean Sea, and the Mediterranean Sea in comparison to Black Sea. There is no information on Mn concentration in fish samples according to Turkish standards [28]. USA National Sciences Academy (1980) recommends daily 2.5–5 mg per person as the amount of Mn, and daily tolerable amount of manganese based on body weight (according to body weight of 60 kg) was suggested as 2–9 mg by WHO [14].

### 3.8. Nickel

According to investigations, plant based nutrients have higher amounts of nickel (Ni) than animal-based nutrients. Compared to other transition metals, Ni is a moderately toxic element. Consumption of food with high Ni content may cause serious problems in observation of many diseases [38]. Inherent amount of Ni in water is rather low, and in studies performed in the USA, such amount was determined as 4.8**μ**g L^−1^. The lowest Ni concentration in fish species was 0.01* µ*g g^−1^ in* Sarda sarda Belone belone *species taken from Karasu and the highest was 26.1* µ*g g^−1^in* Mugil cephalus *species taken from Çark Stream. In studies by [26, 39] performed in Turkey, average Ni concentration was determined as 1.1–10.2**μ**g g^−1^ and 1.2–3.4**μ**g g^−1^, respectively. There is no information on Ni concentration in fish samples according to Turkish standards [28]. Daily tolerable amount of Ni based on body weight of 60 kg was recommended as 100–300* µ*g by WHO [14].

### 3.9. Strontium

Strontium (Sr) is a widely available element in nature and recommended for osteoporosis treatment. Sr is found in respiratory air, soil, drinking water, and nutrients. Introduction of this element into human body takes place mainly by water and foods [40]. The lowest Sr concentration in fish species was 0.17* µ*g g^−1^ in* Exos lucius *species taken from Sapanca and the highest was 13.5* µ*g g^−1^ in* Capoeta pestai *species taken from Sakarya River. In studies reported in the literature, mean concentration of strontium in fish was found as 1.52–16.2* µ*g g^−1^ [18]. There is no information on maximum Sr concentration in fish samples according to Turkish standards [28].

### 3.10. Zinc

Zinc (Zn) is an important trace element in human metabolism and nutrition and plays a major role in functioning of many biochemical processes. Zn deficiency may cause various conditions and intake of excess Zn may cause side effects [41]. The lowest Zn concentration in fish species was 11.5* µ*g g^−1^ in* Barbus capito* species taken from Pamukova and the highest was 52.9* µ*g g^−1^ in* Capoeta pestai *species taken from Sakarya River. Zn ratio allowed for fish by Turkish Food Codex is 50* µ*g g^−1^ [28]. Fish species in this study did not show higher levels of Zn than the legal limit. Average Zn concentration was found as 11.6–63.5* µ*g g^−1^ in fish studies in Turkey [26]. In other fish studies, [32] reported a Zn concentration of 4.62–14.6* µ*g g^−1^ and [18] reported a Zn concentration of 5.6–16* µ*g g^−1^. But, in some studies, high values of Zn, such as 45.0–60.9* µ*g g^−1^ [42], were also detected. Daily tolerable amount of Zn based on body weight of 60 kg was specified as 60 mg by FAO/WHO [33].

## 4. Conclusions

While trace levels of some metal ions show toxic effects on humans, some of them are necessary for sustaining metabolism of human body, even though high concentrations of all metal ions can pose a threat to human health. Therefore, heavy metal ion levels in food and water consumed by human beings are important. In this study, levels of some elements were determined in various fish species living in lakes, rivers, streams, and sea in Sakarya. Wet and MW digestion techniques were used to prepare the samples for analysis. MW method was chosen as the most appropriate method since it takes less time and causes lesser amount of analyte lost. Trace levels of elements were determined successfully in fish samples using ICP-OES technique. Metal concentrations were generally found to be lower than the limit values specified by Turkish Food Codex (TFC), Food and Agriculture Organization (FAO), and World Health Organization (WHO).

In terms of heavy metal content, it could be concluded that there is no risk in consumption of fish collected from Sakarya region in Sakarya River, Çark Stream, Sapanca Lake, and Western Black Sea.

## Figures and Tables

**Table 1:** Instrumental analytical conditions of element analyses.

Instrument	Conditions
ICP-OES	
RF power	1450 W
Auxiliary argon flow rate	0.7 L min^−1^
Plasma gas flow	13 L min^−1^
Nebulizer flow	0.8 L min^−1^
Nebulizer	Modified Lichte
Spray chamber	Cyclonic
Sample aspiration rate	2.0 mL min^−1^
Sample pump rate	25 rpm

**Table 2:** Certified and observed values (*μ*g g^−1^) of trace elements in DORM-3, (*N* = 4).

Element	Certified value	Microvave digestion	Recovery (%)	Wet ashing	Recovery (%)
Al	—	—	—	—	—
B	—	—	—	—	—
Cu	15.5 ± 0.63	14.9 ± 0.23	96	14.3 ± 0.26	92
Cr	1.89 ± 0.17	1.80 ± 0.11	95	1.78 ± 0.14	94
Fe	347 ± 20	338 ± 12	97	330 ± 16	95
Mn	—	—	—	—	—
Ni	1.28 ± 0.24	1.21 ± 0.17	95	1.19 ± 0.19	93
Zn	51.3 ± 3.1	50.5 ± 2.8	98	48.7 ± 3.1	95

**Table 3:** Trace metal contents (*μ*g g^−1^) with microvave and wet ashing methods in *Silurus glanis* sample (mean ± S.D., *N* = 4).

Methods	Concentrations									
	Al	B	Ba	Cr	Cu	Fe	Mn	Ni	Sr	Zn
Microvave	15.9 ± 1.4	0.20 ± 0.05	0.44 ± 0.03	1.64 ± 0.05	0.35 ± 0.05	26.5 ± 1.3	0.33 ± 0.03	0.70 ± 0.05	0.80 ± 0.04	12.9 ± 2.1
Wet ashing	15.1 ± 0.5	0.17 ± 0.02	0.39 ± 0.01	1.58 ± 0.02	0.31 ± 0.03	25.7 ± 1.5	0.30 ± 0.02	0.66 ± 0.04	0.75 ± 0.05	12.2 ± 2.5

**Table 4:** Trace element contents (*μ*g g^−1^) in fish samples after microwave digestion (*N* = 3).

Sample place		Fish species	Elements									
			Al	B	Ba	Cr	Cu	Fe	Mn	Ni	Sr	Zn
Sapanca Lake	Sapanca	*Esox lucius *	48.5 ± 2.1	2.16 ± 0.03	BLD	0.28 ± 0.07	0.34 ± 0.06	26.8 ± 3.1	0.23 ± 0.03	0.58 ± 0.05	0.17 ± 0.03	22.3 ± 1.2
		*Cyprinus carpio *	14.3 ± 1.1	0.40 ± 0.05	BLD	0.08 ± 0.02	1.12 ± 0.07	22.3 ± 1.7	0.38 ± 0.06	0.52 ± 0.06	1.44 ± 0.02	23.1 ± 2.5
		*Scardinius erythrophtholmus *	27.2 ± 2.4	0.88 ± 0.15	0.20 ± 0.02	0.12 ± 0.08	0.66 ± 0.03	21.1 ± 2.3	0.57 ± 0.10	0.19 ± 0.02	1.64 ± 0.05	25.7 ± 2.4
		*Tinca tinca *	6.50 ± 0.07	BLD	BLD	0.26 ± 0.05	0.65 ± 0.07	12.2 ± 1.2	BLD	BLD	0.52 ± 0.04	12.6 ± 1.7

Sakarya River	Sakarya	*Silurus glanis *	15.9 ± 1.4	0.20 ± 0.05	0.44 ± 0.03	1.64 ± 0.05	0.35 ± 0.05	26.5 ± 1.3	0.33 ± 0.03	0.70 ± 0.05	0.80 ± 0.04	12.9 ± 2.1
		*Blicca bjoerkna*	9.85 ± 0.12	3.30 ± 0.1	1.24 ± 0.04	0.08 ± 0.02	1.10 ± 0.04	11.6 ± 0.7	1.06 ± 0.02	0.44 ± 0.01	12.3 ± 1.1	23.8 ± 0.4
		*Capoeta pestai *	13.5 ± 1.1	0.80 ± 0.03	0.15 ± 0.02	0.12 ± 0.01	1.08 ± 0.03	12.5 ± 0.4	1.64 ± 0.04	0.30 ± 0.03	13.5 ± 1.5	52.9 ± 5.2
		*Cyprinus carpio *	23.6 ± 2.1	0.06 ± 0.01	0.09 ± 0.01	0.24 ± 0.03	1.14 ± 0.07	25.4 ± 1.1	11.4 ± 0.1	0.16 ± 0.02	11.1 ± 0.5	45.7 ± 2.0
	Pamukova	*Cyprinus carpio *	9.24 ± 0.12	0.52 ± 0.03	BLD	0.04 ± 0.01	0.13 ± 0.06	18.5 ± 1.2	0.65 ± 0.03	0.26 ± 0.01	3.52 ± 0.04	21.3 ± 1.4
		*Scardinius erythrophtholmus *	7.76 ± 0.18	3.13 ± 0.12	0.57 ± 0.03	0.12 ± 0.03	0.22 ± 0.04	7.28 ± 1.7	BLD	BLD	2.84 ± 0.07	12.9 ± 0.7
		*Mugil cepholus *	10.3 ± 1.1	0.92 ± 0.04	1.42 ± 0.02	0.14 ± 0.06	0.60 ± 0.05	10.8 ± 0.6	0.24 ± 0.04	0.08 ± 0.02	2.68 ± 0.03	16.2 ± 0.4
		*Barbus capito *	23.6 ± 0.5	0.40 ± 0.02	BLD	0.02 ± 0.01	0.92 ± 0.12	12.5 ± 0.5	0.08 ± 0.03	11.6 ± 0.1	7.48 ± 1.12	11.5 ± 1.5

Çark Stream	Sakarya	*Blicca bjoerkna *	11.9 ± 1.2	0.08 ± 0.02	2.92 ± 0.03	0.84 ± 0.04	1.24 ± 0.04	16.1 ± 1.3	1.00 ± 0.03	23.4 ± 3.1	10.1 ± 1.0	13.1 ± 0.6
		*Mugil cepholus *	12.5 ± 0.4	1.12 ± 0.03	BLD	0.16 ± 0.02	1.48 ± 0.03	23.7 ± 2.5	0.56 ± 0.07	26.1 ± 1.2	7.95 ± 0.41	24.0 ± 2.6
		*Scardinius erythrophtholmus *	9.40 ± 1.17	0.20 ± 0.01	0.60 ± 0.02	0.04 ± 0.02	0.80 ± 0.07	17.9 ± 1.2	1.01 ± 0.06	0.16 ± 0.04	2.88 ± 0.03	18.8 ± 2.1

West Black Sea	Karasu	*Trachurus mediterraneus *	10.0 ± 1.1	0.92 ± 0.03	BLD	0.72 ± 0.04	1.52 ± 0.05	39.9 ± 2.5	BLD	0.84 ± 0.05	6.04 ± 0.05	40.4 ± 4.2
		*Sadra sarda *	8.80 ± 1.21	0.60 ± 0.07	BLD	1.32 ± 0.08	1.12 ± 0.02	26.6 ± 1.4	BLD	0.01 ± 0.01	1.42 ± 0.02	12.9 ± 1.5
		*Mullus barbatus *	11.9 ± 2.5	0.36 ± 0.05	BLD	0.16 ± 0.01	2.28 ± 0.03	17.4 ± 0.5	0.08 ± 0.02	0.20 ± 0.03	7.68 ± 0.04	36.4 ± 3.2
		*Engraulis encrasicolus *	9.92 ± 2.32	0.28 ± 0.03	BLD	0.14 ± 0.06	1.34 ± 0.04	25.0 ± 3.9	0.76 ± 0.09	0.34 ± 0.07	3.52 ± 0.02	37.6 ± 0.7
		*Gobius niger *	19.2 ± 3.0	0.50 ± 0.08	1.28 ± 0.02	0.20 ± 0.04	1.16 ± 0.05	28.4 ± 2.1	0.40 ± 0.01	0.36 ± 0.08	1.12 ± 0.03	20.6 ± 1.8
		*Merlangius merlangus *	8.20 ± 1.31	0.22 ± 0.01	BLD	0.24 ± 0.07	1.28 ± .0.07	11.7 ± 1.0	BLD	0.35 ± 0.03	5.64 ± 0.04	18.1 ± 0.3
		*Belone belone *	12.3 ± 2.5	0.48 ± 0.04	BLD	0.04 ± 0.02	1.08 ± 0.01	21.8 ± 1.4	0.57 ± 0.03	0.01 ± 0.01	6.48 ± 0.09	29.3 ± 2.6
		*Pamatomus saltarix *	7.84 ± 1.14	0.25 ± .0.02	0.59 ± 0.02	0.12 ± 0.01	0.33 ± 0.04	21.7 ± 2.7	0.48 ± 0.04	0.84 ± 0.04	0.84 ± 0.07	18.1 ± 0.5
